# Ten years of tracking mental health in refugee primary health care settings: an updated analysis of data from UNHCR’s Health Information System (2009–2018)

**DOI:** 10.1186/s12916-022-02371-8

**Published:** 2022-05-16

**Authors:** Shoshanna L. Fine, Jeremy C. Kane, Paul B. Spiegel, Wietse A. Tol, Peter Ventevogel

**Affiliations:** 1 Department of Population, Family and Reproductive Health, Johns Hopkins Bloomberg School of Public Health, 615 N. Wolfe St, Baltimore, MD 21205 USA; 2 Department of Mental Health, Johns Hopkins Bloomberg School of Public Health, Baltimore, MD USA; 3 Department of Epidemiology, Columbia Mailman School of Public Health, New York, NY USA; 4 Department of International Health, Johns Hopkins Bloomberg School of Public Health, Baltimore, MD USA; 5 Department of Public Health, University of Copenhagen, Copenhagen, Denmark; 6 Athena Research Institute, Vrije Universiteit Amsterdam, Amsterdam, the Netherlands; 7 Arq International, Diemen, the Netherlands; 8 Public Health Section, Division of Resilience and Solutions, United Nations High Commissioner for Refugees, Geneva, Switzerland

**Keywords:** Refugee, Refugee camps, Service utilization, Health information system, Global mental health

## Abstract

**Background:**

This study examines mental, neurological, and substance use (MNS) service usage within refugee camp primary health care facilities in low- and middle-income countries (LMICs) by analyzing surveillance data from the United Nations High Commissioner for Refugees Health Information System (HIS). Such information is crucial for efforts to strengthen MNS services in primary health care settings for refugees in LMICs.

**Methods:**

Data on 744,036 MNS visits were collected from 175 refugee camps across 24 countries between 2009 and 2018. The HIS documented primary health care visits for seven MNS categories: epilepsy/seizures, alcohol/substance use disorders, mental retardation/intellectual disability, psychotic disorders, severe emotional disorders, medically unexplained somatic complaints, and other psychological complaints. Combined data were stratified by 2-year period, country, sex, and age group. These data were then integrated with camp population data to generate MNS service utilization rates, calculated as MNS visits per 1000 persons per month.

**Results:**

MNS service utilization rates remained broadly consistent throughout the 10-year period, with rates across all camps hovering around 2–3 visits per 1000 persons per month. The largest proportion of MNS visits were attributable to epilepsy/seizures (44.4%) and psychotic disorders (21.8%). There were wide variations in MNS service utilization rates and few consistent patterns over time at the country level. Across the 10 years, females had higher MNS service utilization rates than males, and rates were lower among children under five compared to those five and older.

**Conclusions:**

Despite increased efforts to integrate MNS services into refugee primary health care settings over the past 10 years, there does not appear to be an increase in overall service utilization rates for MNS disorders within these settings. Healthcare service utilization rates are particularly low for common mental disorders such as depression, anxiety, post-traumatic stress disorder, and substance use. This may be related to different health-seeking behaviors for these disorders and because psychological services are often offered outside of formal health settings and consequently do not report to the HIS. Sustained and equitable investment to improve identification and holistic management of MNS disorders in refugee settings should remain a priority.

**Supplementary Information:**

The online version contains supplementary material available at 10.1186/s12916-022-02371-8.

## Background

The past decade has seen unprecedented growth in the number of refugees worldwide, with an estimated 82.4 million individuals forcibly displaced by conflict, violence, and persecution as of 2020 [1]. A robust body of literature has documented elevated rates of common mental disorders among refugees and other forcibly displaced populations, including depression, anxiety, and post-traumatic stress disorder (PTSD) [2]. A recent systematic review of mental health among conflict-affected populations found that more than one in five (22.1%) people living in humanitarian settings suffer from a mental disorder [6]. This estimate is considerably higher than the global average [2–5], highlighting the need to strengthen mental health interventions in refugee contexts.

While there is extensive evidence documenting the epidemiology of these common mental disorders among refugees, there is much less information about the full spectrum of mental, neurological, and substance use (MNS) problems, including epilepsy, psychotic disorders, and substance use disorders [7]. This represents an important gap, as existing studies have suggested the salience of such MNS problems in humanitarian environments. The burden of epilepsy is substantially higher in low- and middle-income countries (LMICs) [8], and existing treatment gaps in these settings may be further pronounced in populations affected by conflict and forced displacement [13]. Psychotic disorders have been found to be particularly disabling in humanitarian environments [9, 10], and there is emerging evidence that refugees may have an elevated risk of developing psychosis [11, 12] and may also be vulnerable to PTSD with secondary psychotic features [14, 15]. Finally, there is some indication that substance use disorders are more prevalent among forcibly displaced populations, although few studies have been conducted among refugees living in LMICs [16, 17].

Over the past few decades, such findings have spurred efforts to improve mental health coverage among refugee populations [18, 19]. These include the widespread adoption of the Inter-Agency Standing Committee (IASC) Guidelines for Mental Health and Psychosocial Support in Emergency Settings, which are intended to support multisectoral humanitarian actors in planning and managing coordinated responses for addressing mental health and psychosocial well-being during complex emergencies [20]. In addition, the World Health Organization (WHO) and the United Nations High Commissioner for Refugees (UNHCR) promote the use of their Humanitarian Intervention Guide (HIG) as part of the Mental Health Gap Action Programme (mhGAP), which aims to reduce the global treatment gap for MNS conditions [21]. The mhGAP-HIG offers evidence-based practices to aid non-specialist providers in assessing and treating common MNS disorders among conflict-affected populations and has been used to build MNS capacity in a range of humanitarian environments [22–26].

A critical component in efforts to scale up mental health programs in refugee settings is the routine collection of data on MNS service usage [27]. Such data can be used to identify treatment gaps for particular populations and conditions, guide resource allocation, and inform evidence-based policies and programs targeting MNS problems in these complex environments [28]. In response to the need for routine collection of MNS service information, UNHCR began including MNS indicators in its existing health information system (HIS) in 2009. The HIS captures ongoing data on contact with primary health care services in refugee camps [29]. An analysis of HIS data collected between January 2009 and March 2013 examined MNS service usage in 90 refugee camps and found that while rates were extremely variable across countries, the highest proportion of overall visits were attributable to epilepsy/seizures (40.6%) and psychotic disorders (22.7%) [30]. The authors concluded that (1) refugee primary health care systems must be better equipped to manage severe neuropsychiatric problems and (2) they were likely missing common mental disorders (i.e., depression, anxiety, and PTSD) given the relatively low visit rates for these issues. Furthermore, they suggested that the marked disparities observed across countries may be attributable to an insufficient capacity to identify and treat MNS disorders among providers in some settings.

The current study expands on this previous work by incorporating nearly 6 years of additional data (through December 2018). There have been several important changes since the original publication which warrant this updated analysis. First, there have been unprecedented increases in the global population of refugees over the past decade. For instance, between 2012 and 2018, the number of refugees more than doubled in Ethiopia (from 356,000 to 870,000) and more than quadrupled in Uganda (from 225,000 to 1.19 million) [31]. This influx of new refugees may have variable rates of MNS disorders due to differential exposures to specific risk factors: for example, the outbreak of violence in the Central African Republic (CAR) in 2013 has forced thousands of people into neighboring countries, and ethnic conflict in Myanmar triggered a mass exodus of Rohingya refugees to Bangladesh in 2017 [32]. Second, since the original publication, UNHCR has expanded the HIS into additional countries, including Burkina Faso, Cameroon, CAR, the Democratic Republic of Congo (DRC), the Republic of Congo, Sudan, and South Sudan. Finally, with the publication of the mhGAP-HIG in 2015, UNHCR and its partners have intensified capacity building for the identification and management of MNS problems within refugee primary health care settings.

The aim of this study was to examine MNS service usage within primary health care settings since the initiation of the MNS HIS in 2009, and to explore the extent to which this has changed over the past 10 years. It is important to note that in January 2019, UNHCR gradually introduced a new tablet-based system, the integrated Refugee Health Information System (iRHIS), which has several updated features that were not available in the old HIS. The iRHIS improvements make it challenging to compare data between the old and new systems. As such, the current study is only able to report on data collected through the end of December 2018.

## Methods

### Setting

The present study is a secondary analysis of MNS data collected through the HIS from January 2009 through December 2018. It includes 175 refugee camps in 24 participating countries representing three UNHCR regions. These comprise (1) Africa (Burkina Faso, Burundi, Cameroon, CAR, Chad, DRC, Djibouti, Eritrea, Ethiopia, Ghana, Kenya, Liberia, Namibia, Republic of the Congo, Rwanda, South Sudan, Sudan, Tanzania, Uganda, Zambia); (2) Asia and the Pacific (Bangladesh, Nepal, Thailand); and (3) Middle East and North Africa (Yemen). Importantly, most countries in the Middle East document consultations of refugees to health services through national health information systems, which generally collect limited MNS data. Consequently, this study was unable to include data from Iraq, Jordan, Lebanon, and Turkey, which host the most Syrian and Iraqi refugees, and Iran and Pakistan, which host millions of refugees from Afghanistan.

### Data collection

HIS data were collected within each refugee camp through outpatient primary health care facilities and were entered onto standardized reporting forms by clinicians (see Supplemental Material). These forms included seven MNS categories, which were developed through consultation with mental health experts from the WHO and international non-governmental organizations [29]. Categories were intended to capture the most important mental health issues in humanitarian settings and included (1) epilepsy/seizures, (2) alcohol/substance use disorders, (3) mental retardation/intellectual disability, (4) psychotic disorders, (5) severe emotional disorders (including depression and PTSD), (6) medically unexplained somatic complaints, and (7) other psychological complaints. Case definitions were based on the IASC Guidelines [20]. They were developed to suggest a probable diagnosis in the absence of detailed diagnostic procedures and did not necessarily conform with international classification systems (Table 1). These case definitions were purposely left broad, to make them easy to use by non-specialists working in primary health care settings. For example, *other psychological complaints* were designed to capture general psychological distress comprising emotional (e.g., depressed mood, anxiety), cognitive (e.g., rumination, poor concentration), or behavioral (e.g., inactivity, aggression) symptoms. The HIS standardized reporting form stratified cases by sex and age but did not distinguish between new and revisit cases.

**Table 1:** HIS case definitions for mental, neurological, and substance use disorders

Disorder	Case definition
Epilepsy/seizures	At least two episodes of seizures not provoked by any apparent cause such as fever, infection, injury, or alcohol withdrawal. Episodes are characterized by loss of consciousness with shaking of limbs, and sometimes associated with physical injuries, bowel/bladder incontinence, and tongue biting.
Alcohol/substance use disorder	Consumption of alcohol (or other addictive substances) on a daily basis with difficulties controlling consumption. Personal relationships, work performance, and physical health often deteriorate but consumption continues despite these problems.
Mental retardation/intellectual disability	Very low intelligence causing problems in daily living. As a child, this person is slow in learning to speak. As an adult, the person can work if tasks are simple. This person will rarely be able to live independently or look after themselves/children without support from others. When severe, this person may have difficulties speaking and understanding others and may require constant assistance.
Psychotic disorder	Hearing or seeing things that are not there, or strongly believing things that are not true. This person may talk to themselves, their speech may be confused or incoherent, and their appearance unusual. They may neglect themselves, but may also go through periods of being extremely happy, irritable, energetic, talkative, and reckless. This person’s behavior is considered “crazy” or “highly bizarre” by others from the same culture.
Severe emotional disorder	Daily functioning is markedly impaired for more than 2 weeks due to (a) overwhelming sadness/apathy and/or (b) exaggerated, uncontrollable anxiety/fear. Personal relationships, appetite, sleep, and concentration are often affected. The person may be unable to initiate or maintain conversations. The person may complain of severe fatigue and be socially withdrawn, often staying in bed for much of the day. Suicidal thinking is common.
Medical unexplained somatic complaint	Any somatic/physical complaint that does not have an apparent organic cause. Should only be applied (a) after conducting necessary physical examinations, (b) if the person is not positive for any of the other categories, and (c) if the person is requesting help for the complaint.
Other psychological complaint	This category covers complaints related to emotions (e.g., depressed mood, anxiety), thoughts (e.g., ruminating, poor concentration) or behaviors (e.g., inactivity, aggression). The person tends to be able to function in all or almost all activities of daily living. The complaint may be a symptom of a less severe emotional disorder or may represent normal distress not associated with a disorder.

### Analysis

Data from all 175 refugee camps were combined and were then stratified by 2-year periods (2009–2010, 2011–2012, 2013–2014, 2015–2016, 2017–2018), country, sex, and age group (children younger than 5 years versus individuals 5 years and older). The proportion of MNS problems attributable to each of the seven categories across the entire 10-year study period was calculated. In addition, the service utilization rate for each of the MNS problems was estimated, calculated as the rate of those receiving services relative to the total camp population. Notably, this rate does not include the underlying mental health burden in these refugee camps and therefore cannot be used to assess gaps in coverage between those who need versus those who are accessing services. In the absence of robust prevalence information, however, it has been suggested that service utilization rates are advantageous for service planning, tracking changes over time, and making comparisons between different settings, especially if there is some preexisting knowledge regarding the health burden from epidemiological studies [33].

Category-specific and overall MNS service utilization rates for each 2-year period were first estimated at the camp level. Camp-level rates were calculated by dividing the total number of visits in each MNS category within a given 2-year period by the total number of person-time contributed by the camp in the same period. Person-time reflected the camp’s monthly population during each month that the HIS was active in the 2-year period. The ensuing rate was then multiplied by 1000 to yield MNS visits per 1000 refugees per month for the camp. Weighted mean rates and standard deviations were calculated at the country level, as well as by sex and age categories. Country-level weights were calculated as the ratio of a camp’s contributed person-time to all camps’ contributed person-time within a country. Sex- and age-specific weights were calculated as the ratio of a camp’s contributed person-time to all camps’ contributed person-time within the HIS. In each case, the weighted camp rates were summed to produce the final weighted mean rates: across all camps in a country for the country-level rates, and across all camps in all countries in the HIS for the sex- and age-specific rates. Population estimates used in the calculation of these rates were extracted from a separate HIS population database. All analyses were conducted using Stata 14.2 [34].

## Results

The HIS captured information from an increasing number of refugees over the course of the 10-year study period, starting with a total population of 338,349 from 14 participating camps in January 2009 and ending with a total population of 3,775,658 from 114 participating camps in December 2018. During this time, there were a total of 744,036 reported visits for any MNS disorder: 38,469 visits in 2009–2010, 116,354 visits in 2011–2012, 134,662 visits in 2013–2014, 196,528 visits in 2015–2016, and 258,023 visits in 2017–2018. The weighted mean service utilization rates across all camps in terms of visits per 1000 persons per month were 2.06 in 2009–2010 (SD = 2.35), 3.05 in 2011–2012 (SD = 3.20), 2.46 in 2013–2014 (SD = 2.93), 2.67 in 2015–2016 (SD = 2.95), and 2.68 in 2017–2018 (SD = 3.26). Of the overall visits during this period, most were due to epilepsy/seizures (44.4%), followed by psychotic disorders (21.8%), and severe emotional disorders (11.6%). The smallest number of visits was due to alcohol/substance use disorders (2.2%), mental retardation/intellectual disability (3.7%), medically unexplained somatic complaints (7.4%), and other psychological complaints (9.0%).

### Country

The weighted mean service utilization rates of MNS visits per 1000 refugees per month for each country and MNS category within 2-year periods are presented in Table 2. In 2009–2010, these weighted mean rates ranged from 0.00 in Sudan to 11.13 in Nepal; in 2011–2012, they ranged from 0.00 in Burkina Faso, the Republic of the Congo, Sudan, and South Sudan to 26.28 in Liberia (SD = 13.80); in 2013–2014, they ranged from 0.00 in Cameroon and Sudan to 14.04 in Nepal (SD = 1.20); in 2015–2016, they ranged from 0.00 in CAR and the Republic of the Congo to 21.40 in Nepal (SD = 1.02); and in 2017–2018, they ranged from 0.84 in South Sudan (SD = 1.30) to 22.40 in Nepal (SD = 5.37). Nepal, Liberia, and Burundi consistently had the highest weighted mean rates of total reported MNS visits across all 2-year periods. Conversely, Bangladesh, Burkina Faso, Cameroon, CAR, the Republic of the Congo, Eritrea, Ghana, South Sudan, Sudan, and Zambia consistently had the lowest weighted mean rates of total reported MNS visits across all of the 2-year periods (i.e., rates of less than 1.00 visit per 1000 refugees per month).

**Table 2:** Weighted mean rates of MNS visits per 1000 refugees per month for each participating HIS country from 2009 to 2018

Country (number of camps)	Years	Epilepsy/seizures	Alcohol/substance	Intellectual disability	Psychotic disorder	Emotional disorder	Somatic complaint	Other complaint	Total
		Weighted mean visit rate per 1000 per month (weighted SD)^a^							
Bangladesh (5)	2009–2010	0.01 (0.01)	0.00 (0.00)	0.004 (0.005)	0.04 (0.04)	0.003 (0.01)	0.001 (0.002)	0.03 (0.03)	0.08 (0.07)
	2011–2012	0.08 (0.06)	0.001 (0.001)	0.09 (0.12)	0.05 (0.03)	0.06 (0.07)	0.02 (0.02)	0.06 (0.06)	0.35 (0.35)
	2013–2014	0.07 (0.04)	0.00 (0.00)	0.04 (0.05)	0.13 (0.12)	0.14 (0.18)	0.02 (0.01)	0.12 (0.17)	0.53 (0.57)
	2015–2016	0.25 (0.02)	0.01 (0.01)	0.02 (0.003)	0.16 (0.04)	0.01 (0.01)	0.08 (0.02)	0.21 (0.07)	0.74 (0.07)
	2017–2018	0.24 (0.18)	0.01 (0.01)	0.07 (0.04)	0.20 (0.14)	0.19 (0.13)	0.13 (0.09)	0.29 (0.19)	1.13 (0.50)
Burkina Faso (2)	2011–2012	0.00 (0.00)	0.00 (0.00)	0.00 (0.00)	0.00 (0.00)	0.00 (0.00)	0.00 (0.00)	0.00 (0.00)	0.00 (0.00)
	2013–2014	0.06 (0.04)	0.00 (0.00)	0.002 (0.002)	0.05 (0.05)	0.00 (0.00)	0.00 (0.00)	0.03 (0.03)	0.14 (0.12)
	2015–2016	0.26 (0.15)	0.00 (0.00)	0.02 (0.01)	0.28 (0.10)	0.02 (0.02)	0.01 (0.01)	0.06 (0.04)	0.66 (0.34)
	2017–2018	0.11 (0.02)	0.00 (0.00)	0.01 (0.01)	0.29 (0.17)	0.00 (0.00)	0.01 (0.005)	0.07 (0.05)	0.49 (0.24)
Burundi (4)	2011–2012	5.50 (0.69)	0.11 (0.07)	1.00 (0.73)	3.03 (1.02)	0.83 (0.86)	0.71 (0.61)	1.76 (1.15)	12.94 (1.68)
	2013–2014	4.67 (1.53)	0.02 (0.02)	1.00 (0.75)	2.36 (0.71)	0.61 (0.74)	0.17 (0.13)	0.97 (0.66)	9.81 (3.14)
	2015–2016	4.47 (1.22)	0.04 (0.02)	1.01 (0.45)	1.86 (0.18)	0.44 (0.69)	0.32 (0.17)	0.80 (0.26)	8.93 (1.81)
	2017–2018	2.40 (1.15)	0.04 (0.02)	0.54 (0.15)	0.97 (0.60)	0.27 (0.46)	0.05 (0.05)	0.40 (0.25)	4.67 (2.21)
Cameroon (17)	2009–2010	0.00 (0.00)	0.02 (0.03)	0.005 (0.01)	0.01 (0.02)	0.04 (0.06)	0.01 (0.02)	0.01 (0.02)	0.11 (0.15)
	2011–2012	0.00 (0.00)	0.00 (0.00)	0.00 (0.00)	0.001 (0.01)	0.002 (0.02)	0.00 (0.00)	0.00 (0.00)	0.002 (0.03)
	2013–2014	0.00 (0.00)	0.00 (0.00)	0.00 (0.00)	0.00 (0.00)	0.00 (0.00)	0.00 (0.00)	0.00 (0.00)	0.00 (0.00)
	2015–2016	0.02 (0.02)	0.01 (0.01)	0.00 (0.00)	0.01 (0.02)	0.01 (0.02)	0.004 (0.01)	0.003 (0.01)	0.05 (0.05)
	2017–2018	0.23 (0.17)	0.03 (0.02)	0.00 (0.00)	0.30 (0.21)	0.29 (0.21)	0.08 (0.06)	0.06 (0.05)	1.01 (0.72)
CAR (3)	2011–2012	0.04 (0.05)	0.00 (0.00)	0.00 (0.00)	0.04 (0.05)	0.00 (0.00)	0.00 (0.00)	0.04 (0.05)	0.12 (0.14)
	2015–2016	0.00	0.00	0.00	0.00	0.00	0.00	0.00	0.00
Chad (31)	2009–2010	0.48 (0.67)	0.05 (0.07)	0.06 (0.12)	0.12 (0.20)	0.19 (0.38)	0.01 (0.03)	0.35 (0.36)	1.27 (1.41)
	2011–2012	1.00 (0.74)	0.05 (0.10)	0.15 (0.15)	0.46 (0.32)	0.29 (0.27)	0.09 (0.32)	0.10 (0.08)	2.13 (1.52)
	2013–2014	1.01 (0.71)	0.06 (0.05)	0.15 (0.14)	0.40 (0.29)	0.17 (0.19)	0.06 (0.06)	0.08 (0.08)	1.93 (1.18)
	2015–2016	2.01 (1.56)	0.07 (0.10)	0.26 (0.47)	0.61 (0.41)	0.27 (0.52)	0.09 (0.14)	0.11 (0.11)	3.40 (2.00)
	2017–2018	1.98 (1.18)	0.08 (0.12)	0.24 (0.42)	0.72 (0.30)	0.23 (0.37)	0.07 (0.10)	0.17 (0.16)	3.47 (1.43)
DRC (6)	2013–2014	0.04 (0.17)	0.01 (0.02)	0.002 (0.003)	0.03 (0.05)	0.004 (0.01)	0.01 (0.005)	0.00 (0.00)	0.09 (0.21)
	2015–2016	1.34 (0.81)	1.48 (1.24)	0.27 (0.28)	0.31 (0.22)	0.17 (0.21)	0.30 (0.20)	0.78 (0.64)	4.66 (3.12)
	2017–2018	0.88 (0.67)	0.42 (0.51)	0.19 (0.34)	0.35 (0.25)	0.20 (0.31)	0.33 (0.46)	0.46 (0.66)	2.83 (2.93)
Republic of Congo (2)	2011–2012	0.00 (0.00)	0.00 (0.00)	0.00 (0.00)	0.00 (0.00)	0.00 (0.00)	0.00 (0.00)	0.00 (0.00)	0.00 (0.00)
	2013–2014	0.001 (0.001)	0.00 (0.00)	0.00 (0.00)	0.00 (0.00)	0.001 (0.001)	0.00 (0.00)	0.00 (0.00)	0.003 (0.002)
	2015–2016	0.00	0.00	0.00	0.00	0.00	0.00	0.00	0.00
Djibouti (2)	2009–2010	2.58	0.01	0.20	0.56	0.62	0.91	0.43	5.31
	2011–2012	1.57	0.03	0.10	0.37	0.41	1.89	0.50	4.86
	2013–2014	2.22 (0.45)	0.04 (0.004)	0.05 (0.04)	0.13 (0.02)	0.06 (0.01)	1.16 (0.06)	0.19 (0.04)	3.84 (0.52)
	2015–2016	3.01 (0.35)	0.02 (0.01)	0.09 (0.03)	0.29 (0.08)	0.06 (0.05)	1.67 (0.20)	0.37 (0.11)	5.50 (0.66)
Eritrea (1)	2013–2014	0.00	0.00	0.00	0.06	0.00	0.00	0.17	0.23
	2015–2016	0.00	0.00	0.00	0.00	0.00	0.42	0.00	0.42
Ethiopia (28)	2009–2010	0.78 (0.82)	0.04 (0.11)	0.06 (0.16)	0.26 (0.54)	0.11 (0.22)	0.07 (0.22)	0.10 (0.17)	1.41 (1.78)
	2011–2012	0.34 (0.51)	0.02 (0.04)	0.09 (0.21)	0.37 (0.77)	0.09 (0.12)	0.07 (0.10)	0.06 (0.11)	1.03 (1.52)
	2013–2014	0.78 (1.19)	0.01 (0.02)	0.09 (0.26)	0.35 (0.73)	0.10 (0.24)	0.02 (0.03)	0.05 (0.08)	1.39 (2.31)
	2015–2016	0.82 (1.13)	0.01 (0.06)	0.07 (0.26)	0.32 (0.47)	0.18 (0.24)	0.06 (0.16)	0.06 (0.11)	1.52 (2.08)
	2017–2018	0.73 (1.06)	0.02 (0.09)	0.05 (0.16)	0.28 (0.51)	0.16 (0.26)	0.03 (0.09)	0.06 (0.18)	1.33 (1.95)
Ghana (4)	2011–2012	0.12 (0.18)	0.00 (0.00)	0.00 (0.00)	0.09 (0.19)	0.00 (0.00)	0.00 (0.00)	0.12 (0.25)	0.33 (0.44)
	2013–2014	0.005 (0.01)	0.005 (0.01)	0.005 (0.01)	0.01 (0.01)	0.02 (0.04)	0.01 (0.03)	0.00 (0.00)	0.06 (0.10)
	2015–2016	0.15 (0.43)	0.00 (0.00)	0.00 (0.00)	0.00 (0.00)	0.00 (0.00)	0.00 (0.00)	0.00 (0.00)	0.15 (0.43)
Kenya (7)	2009–2010	0.48 (0.42)	0.04 (0.02)	0.08 (0.04)	0.42 (0.21)	0.29 (0.24)	0.11 (0.08)	0.38 (0.34)	1.81 (1.15)
	2011–2012	1.44 (1.10)	0.05 (0.04)	0.12 (0.06)	0.94 (0.39)	0.63 (0.78)	0.20 (0.19)	0.24 (0.23)	3.61 (2.46)
	2013–2014	1.67 (1.28)	0.03 (0.04)	0.07 (0.03)	0.94 (0.50)	0.58 (0.81)	0.28 (0.32)	0.07 (0.07)	3.64 (2.54)
	2015–2016	1.59 (1.15)	0.02 (0.02)	0.06 (0.04)	0.92 (0.65)	0.27 (0.31)	0.27 (0.42)	0.09 (0.09)	3.22 (2.05)
	2017–2018	2.40 (1.38)	0.05 (0.03)	0.13 (0.05)	1.24 (0.83)	0.22 (0.18)	0.30 (0.51)	0.10 (0.13)	4.44 (2.43)
Liberia (3)	2011–2012	2.59 (1.36)	0.82 (0.43)	1.21 (0.64)	2.20 (1.15)	11.97 (6.29)	0.92 (0.48)	6.56 (3.45)	26.28 (13.80)
	2013–2014	3.22 (0.41)	0.51 (0.46)	0.76 (0.78)	0.83 (0.51)	0.99 (0.77)	0.64 (0.50)	0.60 (0.49)	7.55 (3.52)
	2015–2016	6.41 (1.61)	0.39 (0.13)	1.21 (0.93)	0.94 (0.54)	2.05 (0.65)	0.48 (0.36)	0.63 (0.42)	12.10 (1.67)
Namibia (1)	2009–2010	1.31	0.15	0.07	0.87	0.00	0.00	0.00	2.40
	2011–2012	0.77	0.09	0.50	1.05	0.00	0.01	0.00	2.42
	2013–2014	0.60	0.00	0.02	0.96	0.00	0.02	0.09	1.69
Nepal (2)	2009–2010	2.56	0.04	0.22	3.11	0.31	0.09	4.79	11.13
	2011–2012	2.55 (0.54)	0.28 (0.06)	0.19 (0.11)	3.27 (0.41)	0.77 (0.30)	2.76 (0.88)	2.27 (0.34)	12.08 (0.15)
	2013–2014	2.34 (0.59)	0.30 (0.01)	0.13 (0.17)	3.04 (0.14)	1.58 (0.42)	3.53 (0.06)	3.12 (1.62)	14.04 (1.20)
	2015–2016	3.19 (0.38)	1.27 (0.19)	0.10 (0.04)	4.57 (0.71)	3.30 (0.42)	6.31 (0.61)	2.66 (1.32)	21.40 (1.02)
	2017–2018	3.79 (0.51)	1.47 (1.12)	0.27 (0.04)	5.45 (2.25)	3.98 (0.83)	7.45 (2.27)	0.00 (0.00)	22.40 (5.37)
Rwanda (6)	2009–2010	1.34 (1.07)	0.02 (0.02)	0.16 (0.02)	0.59 (0.47)	0.37 (0.04)	0.42 (0.26)	0.42 (0.33)	3.32 (2.22)
	2011–2012	1.26 (0.83)	0.002 (0.002)	0.02 (0.02)	0.79 (0.66)	0.46 (0.52)	0.07 (0.04)	0.50 (0.47)	3.11 (2.50)
	2013–2014	1.80 (0.85)	0.03 (0.02)	0.05 (0.05)	1.11 (0.85)	0.09 (0.07)	0.33 (0.17)	0.40 (0.30)	3.80 (2.19)
	2015–2016	2.15 (0.49)	0.03 (0.02)	0.05 (0.04)	0.84 (0.42)	0.34 (0.23)	0.42 (0.44)	0.37 (0.24)	4.21 (1.18)
	2017–2018	2.83 (0.78)	0.05 (0.05)	0.11 (0.10)	1.03 (0.26)	0.34 (0.11)	0.20 (0.09)	0.53 (0.39)	5.08 (0.80)
South Sudan (13)	2011–2012	0.00 (0.00)	0.00 (0.00)	0.00 (0.00)	0.00 (0.00)	0.00 (0.00)	0.00 (0.00)	0.00 (0.00)	0.00 (0.00)
	2013–2014	0.002 (0.003)	0.00 (0.00)	0.00 (0.00)	0.001 (0.002)	0.0002 (0.001)	0.00 (0.00)	0.03 (0.05)	0.03 (0.05)
	2015–2016	0.38 (0.67)	0.01 (0.01)	0.004 (0.01)	0.12 (0.24)	0.01 (0.03)	0.01 (0.02)	0.04 (0.08)	0.56 (1.02)
	2017–2018	0.54 (0.78)	0.02 (0.04)	0.004 (0.01)	0.11 (0.22)	0.03 (0.05)	0.03 (0.07)	0.11 (0.22)	0.84 (1.30)
Sudan (7)	2009–2010	0.00 (0.00)	0.00 (0.00)	0.00 (0.00)	0.00 (0.00)	0.00 (0.00)	0.00 (0.00)	0.00 (0.00)	0.00 (0.00)
	2011–2012	0.00 (0.00)	0.00 (0.00)	0.00 (0.00)	0.00 (0.00)	0.00 (0.00)	0.00 (0.00)	0.00 (0.00)	0.00 (0.00)
	2013–2014	0.00 (0.00)	0.00 (0.00)	0.00 (0.00)	0.00 (0.00)	0.00 (0.00)	0.00 (0.00)	0.00 (0.00)	0.00 (0.00)
	2015–2016	0.03 (0.08)	0.00 (0.00)	0.01 (0.02)	0.08 (0.23)	0.001 (0.004)	0.00 (0.00)	0.01 (0.01)	0.12 (0.34)
	2017–2018	1.30 (1.02)	0.01 (0.02)	0.09 (0.11)	0.78 (0.70)	0.29 (0.25)	0.02 (0.02)	0.18 (0.19)	2.67 (1.85)
Tanzania (3)	2009–2010	6.12	0.01	0.09	1.27	0.53	0.05	0.03	8.10
	2011–2012	6.54	0.002	0.17	1.58	1.08	0.05	0.02	9.43
	2013–2014	3.99	0.00	0.04	0.98	0.54	0.01	0.01	5.56
	2015–2016	3.13 (1.28)	0.09 (0.19)	0.10 (0.15)	0.91 (0.16)	0.51 (0.22)	0.29 (0.57)	0.08 (0.11)	5.11 (0.55)
	2017–2018	1.59 (0.96)	0.15 (0.16)	0.12 (0.12)	0.71 (0.39)	0.56 (0.28)	0.73 (0.66)	1.37 (1.51)	5.23 (2.16)
Thailand (9)	2009–2010	1.09 (0.73)	0.03 (0.05)	0.01 (0.01)	0.79 (0.41)	0.06 (0.09)	0.06 (0.11)	0.15 (0.15)	2.19 (0.87)
	2011–2012	1.22 (0.55)	0.04 (0.08)	0.12 (0.28)	1.28 (0.88)	0.07 (0.13)	0.13 (0.19)	0.22 (0.35)	3.09 (1.32)
	2013–2014	1.21 (0.48)	0.03 (0.04)	0.06 (0.23)	1.44 (1.04)	0.08 (0.13)	0.18 (0.18)	0.53 (1.08)	3.53 (1.56)
	2015–2016	1.56 (0.53)	0.14 (0.13)	0.07 (0.12)	1.74 (0.91)	0.13 (0.14)	0.19 (0.19)	0.81 (1.27)	4.65 (1.72)
	2017–2018	1.51 (0.57)	0.46 (0.45)	0.16 (0.43)	1.89 (1.08)	0.39 (0.56)	0.37 (0.52)	1.25 (4.59)	6.02 (6.16)
Uganda (14)	2009–2010	0.25 (0.40)	0.02 (0.05)	0.02 (0.03)	0.06 (0.08)	0.09 (0.06)	0.27 (0.16)	0.16 (0.07)	0.87 (0.62)
	2011–2012	0.97 (1.09)	0.03 (0.05)	0.04 (0.06)	0.46 (0.83)	0.26 (0.29)	0.59 (0.29)	0.19 (0.27)	2.54 (2.51)
	2013–2014	0.65 (0.80)	0.03 (0.08)	0.06 (0.10)	0.28 (0.66)	0.21 (0.27)	0.14 (0.25)	0.19 (0.29)	1.57 (2.18)
	2015–2016	0.56 (0.58)	0.06 (0.05)	0.08 (0.08)	0.27 (0.55)	0.25 (0.34)	0.18 (0.23)	0.19 (0.20)	1.59 (1.85)
	2017–2018	0.86 (0.48)	0.06 (0.04)	0.08 (0.08)	0.22 (0.35)	0.24 (0.31)	0.16 (0.30)	0.25 (0.32)	1.87 (1.45)
Yemen (3)	2009–2010	0.67 (0.48)	0.01 (0.01)	0.10 (0.05)	0.92 (0.45)	1.21 (0.66)	0.56 (0.57)	1.45 (1.84)	4.91 (3.29)
	2011–2012	0.74 (0.31)	0.04 (0.03)	0.07 (0.02)	0.82 (0.27)	1.61 (0.57)	0.47 (0.34)	0.63 (0.31)	4.37 (1.67)
	2013–2014	0.67 (0.18)	0.01 (0.01)	0.08 (0.02)	0.92 (0.13)	1.91 (0.42)	0.53 (0.06)	0.22 (0.06)	4.35 (0.58)
	2015–2016	0.91 (0.92)	0.01 (0.01)	0.13 (0.18)	1.01 (0.42)	2.73 (2.68)	0.55 (0.08)	0.48 (0.42)	5.83 (4.38)
	2017–2018	1.67 (2.97)	0.01 (0.02)	0.33 (0.69)	1.58 (1.40)	4.30 (6.80)	0.78 (0.54)	1.22 (1.71)	9.89 (13.86)
Zambia (2)	2009–2010	0.04 (0.03)	0.05 (0.04)	0.005 (0.004)	0.04 (0.04)	0.002 (0.002)	0.03 (0.02)	0.002 (0.003)	0.16 (0.01)
	2011–2012	0.28 (0.24)	0.01 (0.01)	0.00 (0.00)	0.08 (0.05)	0.00 (0.00)	0.01 (0.005)	0.01 (0.01)	0.38 (0.19)
	2013–2014	0.01 (0.01)	0.01 (0.01)	0.00 (0.00)	0.05 (0.04)	0.00 (0.00)	0.00 (0.00)	0.00 (0.00)	0.07 (0.02)
	2015–2016	0.003 (0.003)	0.003 (0.003)	0.00 (0.00)	0.22 (0.20)	0.00 (0.00)	0.02 (0.02)	0.00 (0.00)	0.24 (0.21)

^a^Rates were first calculated at the camp level. For each camp, the numerator of the rate was the total number of visits in each MNS category within a given 2-year period. The denominator was the total number of person-time contributed by the camp in the same period, which reflected the camp’s monthly population during the months that the HIS was active. The resulting rate was multiplied by 1000. For each country, weighted mean rates and standard deviations were calculated from the camp-level rates within that country. Weights were calculated as the ratio of a camp’s contributed person-time to all camp’s contributed person-time within a country. Therefore, the weights summed to 1. *MNS*, mental, neurological, and substance use; *HIS*, health information system

Of the 14 countries with weighted mean rates of greater than 1.00 MNS visit per 1000 refugees per month, there were six in which there was a generally increasing pattern in total reported MNS visits between 2009 and 2019 (Chad, Kenya, Nepal, Rwanda, Thailand, and Yemen). There was one country in which there was a generally decreasing pattern in total reported MNS visits between 2009 and 2019 (Burundi). In the remaining seven countries, there were no clear patterns in total reported MNS visits during this time period (DRC, Djibouti, Ethiopia, Liberia, Namibia, Tanzania, and Uganda). Of the same 14 countries, there were ten in which epilepsy/seizures had the highest rate of all MNS categories across most of the 2-year periods (Burundi, Chad, DRC, Djibouti, Ethiopia, Kenya, Liberia, Rwanda, Tanzania, and Uganda). There were two countries in which psychotic disorders had the highest rates (Namibia and Thailand), one country in which severe emotional disorders had the highest rates (Yemen), and one country in which medically unexplained somatic complaints had the highest rates (Nepal).

### Sex

Table 3 displays the weighted mean MNS service utilization rates separately by sex during the study period. Across all of the 2-year periods, females had higher overall mean MNS service utilization rates per 1000 per month than males: 2.17 (SD = 2.73) compared to 1.94 (SD = 2.06) in 2009–2010, 3.12 (SD = 3.57) compared to 2.98 (SD = 3.00) in 2011–2012, 2.50 (SD = 3.26) compared to 2.42 (SD = 2.80) in 2013–2014, 2.69 (SD = 3.29) compared to 2.64 (SD = 2.77) in 2015–2016, and 2.75 (SD = 3.66) compared to 2.61 (SD = 3.01) in 2017–2018. When broken down by MNS categories, females had higher service utilization rates for severe emotional disorders, medically unexplained somatic complaints, and other psychological complaints across all of the 2-year periods, whereas males had higher service utilization rates for epilepsy/seizures, alcohol/substance use disorders, mental retardation/intellectual disability, and psychotic disorders. These disparities are also reflected in differences between males and females in the proportion of overall visits attributable to each MNS category: epilepsy/seizures (males: 48.9%; females: 40.2%), alcohol/substance use disorders (males: 3.3%; females: 1.1%), mental retardation/intellectual disability (males: 4.1%; females: 3.3%), psychotic disorders (males: 24.2%; females: 19.7%), severe emotional disorders (males: 8.5%, females: 14.5%), medically unexplained somatic complaints (males: 4.9%; females: 9.6%), and other psychological complaints (males: 6.2%; females: 11.6%). Despite these differences, epilepsy/seizures had the highest service utilization rates for the duration of the study period among both males and females, ranging from 0.83 (SD = 1.26) to 1.37 (SD = 1.64) among males, and 0.72 (SD = 1.29) to 1.19 (SD = 1.35) among females. Likewise, alcohol/substance use disorders had the lowest service utilization rates, ranging from 0.05 (SD = 0.07) to 0.11 (SD = 0.45) among males, and 0.01 (SD = 0.02) to 0.05 (SD = 0.23) among females.

**Table 3:** Weighted mean rates of MNS visits per 1000 refugees per month by sex and age group from 2009 to 2018

MNS category	Years	Male			Female		
		<5 years old	5 and above	Total	<5 years old	5 and above	Total
		Weighted mean visit rate per 1000 per month (weighted SD)^a^					
Epilepsy/seizures	2009–2010	0.44 (0.86)	0.91 (1.37)	0.83 (1.26)	0.33 (0.71)	0.80 (1.44)	0.72 (1.29)
	2011–2012	0.59 (0.99)	1.53 (1.81)	1.37 (1.64)	0.47 (0.88)	1.24 (1.55)	1.11 (1.42)
	2013–2014	0.48 (0.89)	1.36 (1.62)	1.20 (1.46)	0.45 (0.98)	1.08 (1.25)	0.97 (1.16)
	2015–2016	0.50 (0.96)	1.53 (1.71)	1.34 (1.52)	0.48 (0.93)	1.33 (1.50)	1.19 (1.35)
	2017–2018	0.56 (0.86)	1.44 (1.51)	1.29 (1.36)	0.45 (0.68)	1.28 (1.21)	1.13 (1.09)
Alcohol/substance	2009–2010	0.00 (0.00)	0.06 (0.08)	0.05 (0.07)	0.00 (0.00)	0.01 (0.03)	0.01 (0.02)
	2011–2012	0.003 (0.02)	0.08 (0.14)	0.07 (0.12)	0.001 (0.01)	0.02 (0.04)	0.01 (0.03)
	2013–2014	0.002 (0.03)	0.06 (0.14)	0.05 (0.12)	0.003 (0.05)	0.02 (0.07)	0.02 (0.06)
	2015–2016	0.003 (0.03)	0.14 (0.57)	0.11 (0.45)	0.003 (0.02)	0.06 (0.28)	0.05 (0.23)
	2017–2018	0.002 (0.01)	0.12 (0.28)	0.10 (0.23)	0.004 (0.02)	0.05 (0.17)	0.04 (0.14)
Intellectual disability	2009–2010	0.04 (0.09)	0.07 (0.08)	0.07 (0.08)	0.02 (0.03)	0.04 (0.06)	0.04 (0.06)
	2011–2012	0.11 (0.27)	0.12 (0.17)	0.12 (0.18)	0.06 (0.15)	0.10 (0.21)	0.09 (0.19)
	2013–2014	0.07 (0.29)	0.09 (0.22)	0.09 (0.22)	0.06 (0.25)	0.08 (0.24)	0.08 (0.23)
	2015–2016	0.08 (0.44)	0.12 (0.33)	0.11 (0.30)	0.09 (0.42)	0.10 (0.29)	0.10 (0.26)
	2017–2018	0.10 (0.39)	0.12 (0.26)	0.11 (0.25)	0.09 (0.29)	0.10 (0.21)	0.09 (0.20)
Psychotic disorder	2009–2010	0.01 (0.05)	0.61 (0.65)	0.51 (0.56)	0.01 (0.04)	0.49 (0.60)	0.41 (0.52)
	2011–2012	0.004 (0.01)	1.02 (0.99)	0.84 (0.83)	0.01 (0.02)	0.83 (1.04)	0.69 (0.88)
	2013–2014	0.01 (0.02)	0.80 (0.98)	0.65 (0.83)	0.01 (0.06)	0.66 (0.93)	0.54 (0.78)
	2015–2016	0.01 (0.05)	0.74 (0.89)	0.61 (0.75)	0.01 (0.05)	0.64 (0.85)	0.53 (0.71)
	2017–2018	0.01 (0.02)	0.68 (1.02)	0.56 (0.86)	0.005 (0.02)	0.58 (0.80)	0.48 (0.67)
Emotional disorder	2009–2010	0.02 (0.11)	0.21 (0.27)	0.18 (0.23)	0.01 (0.05)	0.36 (0.59)	0.30 (0.49)
	2011–2012	0.01 (0.06)	0.36 (0.60)	0.30 (0.49)	0.01 (0.05)	0.66 (1.17)	0.55 (0.97)
	2013–2014	0.002 (0.01)	0.26 (0.47)	0.21 (0.38)	0.001 (0.004)	0.49 (0.96)	0.41 (0.82)
	2015–2016	0.003 (0.01)	0.26 (0.53)	0.21 (0.44)	0.002 (0.01)	0.41 (0.98)	0.34 (0.82)
	2017–2018	0.002 (0.01)	0.25 (0.68)	0.21 (0.57)	0.01 (0.02)	0.46 (1.74)	0.38 (1.46)
Somatic complaint	2009–2010	0.003 (0.01)	0.11 (0.17)	0.09 (0.14)	0.004 (0.03)	0.21 (0.41)	0.17 (0.35)
	2011–2012	0.01 (0.03)	0.17 (0.31)	0.14 (0.27)	0.01 (0.04)	0.40 (0.90)	0.33 (0.78)
	2013–2014	0.01 (0.04)	0.14 (0.29)	0.12 (0.24)	0.01 (0.08)	0.31 (0.83)	0.26 (0.71)
	2015–2016	0.01 (0.08)	0.16 (0.36)	0.13 (0.31)	0.02 (0.14)	0.31 (0.97)	0.26 (0.83)
	2017–2018	0.02 (0.07)	0.16 (0.38)	0.13 (0.32)	0.02 (0.08)	0.29 (0.78)	0.24 (0.66)
Other complaint	2009–2010	0.03 (0.07)	0.25 (0.52)	0.21 (0.44)	0.03 (0.06)	0.61 (1.34)	0.52 (1.15)
	2011–2012	0.03 (0.08)	0.17 (0.34)	0.15 (0.29)	0.02 (0.06)	0.39 (0.83)	0.33 (0.71)
	2013–2014	0.02 (0.07)	0.12 (0.28)	0.10 (0.24)	0.01 (0.06)	0.27 (0.97)	0.23 (0.84)
	2015–2016	0.02 (0.11)	0.15 (0.27)	0.13 (0.23)	0.02 (0.09)	0.27 (0.72)	0.23 (0.62)
	2017–2018	0.03 (0.33)	0.25 (0.70)	0.21 (0.60)	0.04 (0.26)	0.45 (1.57)	0.38 (1.33)
**Total**	2009–2010	0.55 (1.00)	2.22 (2.31)	1.94 (2.06)	0.40 (0.76)	2.51 (3.11)	2.17 (2.73)
	2011–2012	0.75 (1.23)	3.46 (3.42)	2.98 (3.00)	0.58 (1.02)	3.63 (4.09)	3.12 (3.57)
	2013–2014	0.60 (1.13)	2.82 (3.20)	2.42 (2.80)	0.55 (1.24)	2.91 (3.71)	2.50 (3.26)
	2015–2016	0.63 (1.31)	3.08 (3.21)	2.64 (2.77)	0.63 (1.28)	3.13 (3.78)	2.69 (3.29)
	2017–2018	0.72 (1.31)	3.01 (3.43)	2.61 (3.01)	0.62 (1.00)	3.20 (4.25)	2.75 (3.66)

^a^Rates were first calculated at the camp level. For each camp, the numerator of the rate was the total number of visits in each MNS category within a given 2-year period within each age/sex category. The denominator was the total number of person-time contributed by the camp in the same period, which reflected the camp’s monthly population during the months that the HIS was active within each age/sex category. The resulting rate was multiplied by 1000. Weighted mean rates and standard deviations were calculated within each age/sex category. Weights were calculated as the ratio of a camp’s contributed person-time to all camp’s contributed person-time within the HIS. Therefore, the weights summed to 1. *MNS*, mental, neurological, and substance use; *HIS*, health information system; *CAR*, Central African Republic; *DRC*, Democratic Republic of Congo

### Children under five

Table 3 shows the weighted mean MNS service utilization rates separately for those younger and older than 5 years. Across all categories, MNS service utilization rates per 1000 per month were lower among children under five, compared to those five and older. For four of the 2-year periods, boys under five had higher overall MNS service utilization rates per 1000 compared to girls under five: 0.55 (SD = 1.00) compared to 0.40 (SD = 0.76) in 2009–2010, 0.75 (SD = 1.23) compared to 0.58 (SD = 1.02) in 2011–2012, 0.60 (SD = 1.13) compared to 0.55 (SD = 1.24) in 2013–2014, and 0.72 (SD = 1.31) compared to 0.62 (SD = 1.00) in 2017–2018. In 2015–2016, the overall MNS service utilization rates were equal between boys (0.63, SD = 1.31) and girls (0.63, SD = 1.28). For both boys and girls under five, epilepsy/seizures had the highest visits rates for the duration of the study period, ranging from 0.44 (SD = 0.86) to 0.59 (SD = 0.99) among boys, and 0.33 (SD = 0.71) to 0.48 (SD = 0.93) among girls. Mental retardation/intellectual disability had the second highest service utilization rates, ranging from 0.04 (SD = 0.09) to 0.11 (SD = 0.27) among boys, and 0.02 (SD = 0.03) to 0.09 (SD = 0.29) among girls. Service utilization rates for alcohol/substance use disorders, psychotic disorders, severe emotional disorders, medically unexplained somatic complaints, and other psychological complaints were negligible for boys and girls under five years.

## Discussion

The current study evaluated MNS service usage within primary health care facilities among refugees living in 175 refugee camps in 24 countries between 2009 and 2018 using UNHCR HIS data. Extending results from a prior study of HIS data, we assessed the service utilization rates for seven MNS problems within 2-year periods, stratified by country, sex, and age. We found that the overall MNS service utilization rates remained consistent during the 10-year period, with weighted mean rates across all camps hovering around 2–3 visits per 1000 persons per month. This suggests that despite a sharp increase in the total population of refugees, UNHCR and its partners were able to maintain a consistent level of MNS support across all refugee camps. While this temporal stability can be seen as an achievement by itself given unprecedented levels of global displacement [32], the average numbers of MNS consultations remain lower than desired and are an indication that MNS disorders may not be adequately addressed within refugee primary health care settings.

Over the last decade, various new tools have been developed with the goal of scaling-up delivery of MNS services through task-sharing approaches aimed at improving mental health coverage among refugee populations (e.g., the mhGAP-HIG) [21] and considerable efforts have been made to train and supervise staff with these methods in some regions [22, 35–37]. An evaluation of mhGAP-HIG capacity building efforts in refugee camps in seven sub-Saharan African countries showed various effects such as (1) strengthened capacities by facility- and community-based staff to deliver mental health and psychosocial support interventions, (2) positive changes in their attitudes towards people suffering from MNS conditions, and (3) improved collaboration among health and non-health staff regarding people suffering from MNS conditions [22]. The authors also remark, however, that capacity building is a “process” and not an “event” and that mhGAP trainings constitute only one element in a spectrum of activities aimed at integrating mental health into primary health care, including regular supervision, continuing on-the-job training, and sufficient human resources. While our data cannot be used to directly evaluate such efforts, in our view, the sustained low MNS service utilization rates speak to the major challenges in integrating mental health services into primary health care in low-resource humanitarian settings due to factors such as staff attrition, lack of sufficient training, lack of supportive clinical supervision, time limitations among primary health care workers, insufficient funding, and variable health-seeking behaviors for MNS problems [38–42].

At the country level, there were very few consistent observed trends in overall MNS service utilization rates over the 10-year period. Within several countries, however, noteworthy patterns emerged. Specifically, within Chad, Kenya, Nepal, Rwanda, Thailand, and Yemen, there were generally increasing patterns in total reported MNS visit rates, whereas in Burundi, there was an overall decreasing pattern. By contrast, MNS visit rates largely remained stable in DRC, Djibouti, Ethiopia, Liberia, Namibia, Tanzania, and Uganda. To further illustrate the types of factors that may underlie these patterns, we have selected three refugee camps with differential results to examine as case studies. All three camps are located in East African countries with significant refugee populations: (1) Hagadera refugee camp in Kenya where MNS service utilization rates showed a gradual increase over the 10-year period, (2) Musasa refugee camp in Burundi where they showed a gradual decrease, and (3) Nakivale refugee settlement in Uganda where they showed no clear pattern. While these case studies cannot provide any conclusive evidence, their purpose is to help contextualize these data and generate hypotheses regarding potential drivers of these differences.

**Fig. 1:**
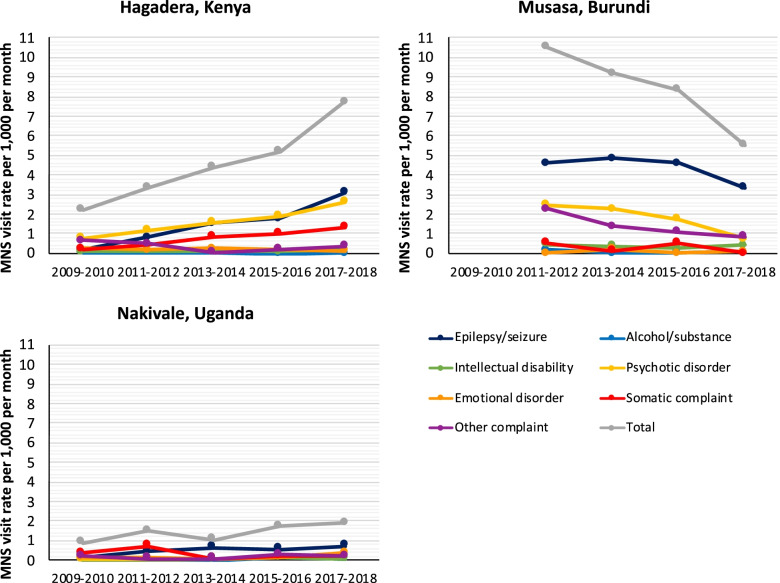
MNS visits per 1000 refugees per month in Hagadera, Musasa, and Nakivale refugee camps from 2009 to 2018

Tables 4 and 5 illustrate the overall MNS visit rates per 1000 per month for the three selected locations alongside the most significant mental health activities that took place from 2009 through 2018. The specific MNS conditions that account for these patterns are further illustrated in Fig. 1. Mental health activities were collected through UNHCR health officers and other health care organizations in the selected camps, who provided annual activity reports, training reports, and other relevant health statistics beyond those collected in the HIS. We examined these documents for information on (1) the organization of mental health services, (2) staffing for mental health activities, (3) training and supervision for mental health activities (e.g., using the mhGAP-HIG), (4) mental health community engagement activities, and (5) intersectoral collaboration with other services providers. While these cases studies are not exhaustive, and findings cannot be generalized to all 175 refugee camps, they provide some interesting observations. Posting a mental health professional in a camp/settlement, which happened in all three sites, is in itself insufficient to explain the substantial camp-level differences. Additional factors, including regular mental health trainings, supportive supervision of primary health care staff, consistent efforts to engage refugee volunteers in mental health work, strong coordination efforts, and robust referral systems with other organizations, seem to be particularly important for success.

**Table 4:** Case studies of Hagadera, Musasa, and Nakivale refugee camps

Refugee camp	Years	MNS visit rate per 1000 per month	Mental health training for general staff	Active community engagement for mental health	Intersectoral collaboration
Hagadera, Kenya	2009–2010	2.22	No	No	Limited
	2011–2012	3.34	No	Yes	Limited
	2013–2014	4.42	Yes	Yes	Intensive
	2015–2016	5.20	Yes	Yes	Intensive
	2017–2018	7.76	Yes	Yes	Intensive
Musasa, Burundi	2009–2010	-	Yes	Yes	Intensive
	2011–2012	10.52	Yes	Yes	Intensive
	2013–2014	9.21	No	No	Limited
	2015–2016	8.34	No	No	Limited
	2017–2018	5.51	No	No	Limited
Nakivale, Uganda	2009–2010	0.90	No	No	Limited
	2011–2012	1.49	No	No	Limited
	2013–2014	1.06	No	No	Limited
	2015–2016	1.72	Yes	No	Limited
	2017–2018	1.89	Yes	No	Intensive

*MNS*, mental, neurological, and substance use

**Table 5:** Mental health activities in Hagadera, Musasa, and Nakivale refugee camps from 2009 to 2018

**Hagadera, Kenya**	***Context*:** Hagadera refugee camp in northeastern Kenya was established in 1992 for Somali refugees. From the onset, mental health services were integrated within the primary health care services delivered by an NGO. Initially, mental health services were concentrated in the camp’s main health center but starting in 2011, satellite mental health clinics were opened in three additional health posts. ***Staffing*:** One psychiatric nurse worked in the camp starting in 2010 and was supported by a team of six trained refugee mental health workers who were actively supervised by the psychiatric nurse. Case identifications and home follow-up visits were conducted by general community health workers. ***Training and supervision****:* Brief mental health trainings were organized annually for health staff. Five-day basic mental health trainings were also organized for mental health workers with the mhGAP-HIG in 2013 and 2018. Clinical supervision was organized by the psychiatric nurse. During weekly trainings for community health workers, mental health was a regular topic. ***Community engagement*:** Every month there were an average of 12 community engagement activities, including meetings with community leaders, youth groups, teachers, religious leaders, and family members of people with severe mental health issues in different parts of the camp. ***Intersectoral collaboration*:** There were close relationships with organizations across sectors including gender-based violence, child protection, and social work, with clear referral pathways. Starting in 2013, a mental health technical working group for all regional camps met regularly.
**Musasa, Burundi**	***Context*:** Musasa refugee camp in northern Burundi was established in 2005. Medical services were provided by an NGO. Until 2009, a specialized NGO provided additional mental health services. After 2009, these were integrated within primary health care services. ***Staffing:*** One nurse was trained in mental health but did not have a formal mental health diploma. There was also one psychologist attached to the clinic. Until 2011, there was a community outreach team of psychosocial volunteers which was discontinued due to budget cuts. ***Training and supervision*:** From 2009 to 2011, a specialized NGO provided a series of mental health trainings for health and protection services staff and refugee volunteers. Health staff were supervised by a physician from the provincial health department. ***Community engagement:*** Until 2012, there were monthly community meetings around mental health, and counselors organized recreational activities for youth and conducted home visits for people with severe mental disorders. These decreased over time due to staff attrition and lack of training for new staff. ***Intersectoral collaboration:*** There were no formal coordination meetings around mental health.
**Nakivale, Uganda**	***Context:*** Nakivale refugee settlement in southwestern Uganda was established in 1958. The settlement is 80 km^2^, with refugees scattered over dozens of “villages.” From 2009 to 2018, one NGO organized health services in seven health facilities. ***Staffing:*** From 2009 to 2014, there was one psychiatric nurse, and from 2015 to 2019, there were two psychiatric nurses. ***Training and supervision:*** In 2017, a mhGAP training of trainers was conducted, followed by a training for primary health care workers. In 2018, a mhGAP training for primary health care workers was conducted. Until 2015, a psychiatric clinical officer from the regional hospital did monthly supervision. ***Community engagement*:** A total of 385 community health workers received trained on mental health in 2018. ***Intersectoral collaboration:*** After 2017, an NGO started providing psychosocial services and a mental health coordination group was established.

*NGO*, non-governmental organization; *mhGAP*, Mental Health Gap Action Programme; *HIG*, Humanitarian Intervention Guide

In terms of specific MNS problems, across most of the countries and among both males and females, we found greater service utilization rates for epilepsy/seizures and psychotic disorders compared to the other MNS problems over the 10-year period, which is consistent with results from the previous HIS study [2] as well as other studies conducted in humanitarian settings [11]. Furthermore, epilepsy/seizures (44.4%) and psychotic disorders (21.8%) were responsible for the highest proportion of overall MNS visits. Although service utilization rates provide no information about underlying prevalence, previous research has suggested that while these types of serious mental and neurological disorders can be immensely disabling in humanitarian settings, they account for a relatively low percentage of overall MNS problems [30]. For instance, according to a recent systematic review, 5.1% of people living in humanitarian settings suffered from a severe mental disorder compared to 17.0% with a mild or moderate mental disorder [2, 7, 11, 12]. As such, the higher service utilization rates for these serious disorders are promising: they indicate that many refugees experiencing these issues are able to find their way to treatment. However, these data do not reflect whether or not care for epilepsy/seizures or psychotic disorders is being sustained over time, suggesting that UNHCR and its partners should prioritize keeping these individuals in ongoing care.

By contrast, there were much lower service utilization rates for common mental disorders, including severe emotional problems, alcohol/substance use disorders, and other psychological complaints. Given the elevated prevalence of these problems in humanitarian settings [2, 3, 17], this overall lack of service utilization within primary health care settings is concerning, particularly given the recent global focus on integrating mental health into primary health care through mhGAP [21]. We hypothesize that this low service utilization in health facilities may relate to differences in illness beliefs and health-seeking behaviors for emotional distress and substance use problems compared to neurological and psychotic disorders. Specifically, if individuals do not view these more common problems as medical issues, they may be less likely to seek care through formal health services and more likely to turn towards informal psychosocial supports within the community. Indeed, one qualitative study conducted among conflict-affected adults in three countries found that whereas symptoms related to psychotic disorders were seen as abnormalities in need of medical treatment, those related to general psychological distress were expected to improve solely through social and emotional support [43]; similar findings have been documented in other LMICs [47]. Overall, this suggests that to improve MNS coverage for refugee populations, it may be important to place an increased emphasis on the availability of non-medicalized, community-based interventions [44–46]. For example, there is a growing body of evidence regarding the effectiveness of brief, psychotherapeutic interventions that target symptoms across a range of common mental health problems and can be delivered by trained non-specialist providers [53–55]. Unfortunately, despite several calls to action, research has lagged in generating evidence around promising approaches for addressing substance use in humanitarian settings [48–52].

Finally, our findings around sex and age differences in MNS service utilization rates remained largely consistent with the previous study. While females had slightly higher service utilization rates compared to males across the 10-year period, there were marked differences in the drivers of these MNS visits. Notably, females were more likely to utilize services for MNS problems related to emotional distress, including severe emotional disorders, medically unexplained somatic complaints, and other psychological complaints; this is consistent with epidemiologic studies drawn from refugee populations [56, 57]. Males were more likely to utilize services for alcohol/substance use disorders, which again aligns with existing literature [16, 17]. Monthly service utilization rates for children under five were negligible for all of the MNS problems besides epilepsy/seizures, which was slightly higher among boys (0.44 per 1000) compared to girls (0.33 per 1000).

### Limitations

This study has several limitations that are important to mention. First, the HIS reporting forms made no differentiation between new and revisit consultations. It was therefore impossible to calculate the incidence rates of MNS problems, limiting the epidemiologic conclusions that can be drawn from these data. We also cannot assume the independence of data collected over different years and are therefore unable to assess the statistical significance of observed trends. Second, the HIS did not capture information on comorbidity despite the high level of co-occurrence among many MNS problems. Third, the HIS included no measure of problem severity. Fourth, whereas the HIS MNS visit data included four age group categories (0–4, 5–17, 18–59, and 60+), available population data only differentiated between those younger and older than 5 years old. As such, we were unable to analyze differences in service utilization rates by these more specific age groups. Fifth, there was substantial variation in terms of how many months the HIS was active across camps. We addressed this, however, by calculating weighted service utilization rates which accounted for the total person-time contributed by each camp.

A final major limitation of HIS data is that they are restricted to the provision of MNS care within primary health care facilities. At its essence, the HIS is a method to record consultations between a patient and a health worker in a health center. Comprehensive mental health and psychosocial support programs within refugee settings consist of a range of activities that take place outside of primary health care facilities and are therefore not captured by the HIS. These frequently include (1) community-based mental health activities, e.g., mental health promotion activities or home visits by community health volunteers; (2) mental health activities conducted by non-health organizations, e.g., school- or faith-based counseling programs; and (3) referrals to nearby health facilities, such as hospitalization in a psychiatric ward of a regional hospital [58–60]. It is also important to note that while health partners that are funded through UNHCR are required to use the HIS to report MNS consultations, those that are funded externally (e.g., Doctors Without Borders) do not consistently use this system. We hypothesize that this important limitation may have contributed to an underestimation in MNS service utilization rates, particularly for common mental health problems which may be more amenable to treatment outside of primary health care facilities (e.g., mild-to-moderate emotional disorders, substance use conditions, other psychological complaints), or in refugee camps where community-based organizations are particularly active [61, 62].

In response to many of the abovementioned limitations, the HIS underwent a significant revision process in 2019, which resulted in several important changes [29]. First, the number of MNS categories was increased from seven to nine, with the addition of “suicide/self-harm” and “dementia/delirium.” The new system also allows for multiple categories to be selected for a single patient at a single consultation and is therefore able to register comorbidity. In addition, it includes an option to add specifiers for trained mental health workers (e.g., psychiatric nurses, mental health outpatient clinicians) to make specific diagnoses when possible. The new system also differentiates between new cases and revisits and includes more refined age categories. Finally, the new system relies on electronic rather than paper data collection, thereby improving data accuracy and timeliness of reporting.

## Conclusions

The findings from this study describe how, overall, MNS service utilization rates in primary health care facilities in refugee camps around the world remained consistent over a 10-year period. Given the enormous increase in the number of global refugees during this time, this can be considered a formidable achievement by itself. It is clear, however, that more significant and sustained efforts are warranted to ensure that refugees in remote and resource-constrained settings can access mental health services. UNHCR’s new *Global Strategy for Public Health 2021–2025* includes the following priority actions to reach this goal [63]:

1. Continued integration of mental health into primary health care facilities for refugees. This includes regularly organizing trainings for primary health care staff in identifying and managing mental health conditions, and arranging for mental health professionals to both treat people with complex conditions and provide clinical supervision to primary health care workers. Efforts towards this action are already underway. In 2021 alone, UNHCR and its partners used the mhGAP-HIG [21] to train 1330 primary health care workers (including doctors, nurses, and medical assistants) in refugee camps across nine countries (DRC, Ethiopia, Jordan, Kenya, Niger, Rwanda, South Sudan, Sudan, and Uganda) (UNHCR Public Health Section, oral communication, March 2022).
2. Provision of evidence-based psychotherapeutic interventions. Not only do primary health care workers within refugee contexts need to be better equipped to address common mental health conditions (e.g., depression, anxiety, PTSD, and substance use), but more also needs to be done to provide treatment and support outside of health facilities. As mentioned previously, the recent surge in research around “scalable psychological interventions” in humanitarian settings provides increasing opportunities to administer brief, evidence-based psychological therapies that can be delivered by trained and supervised non-specialist providers, including refugees themselves [49–51, 64]. Again, efforts towards this action are ongoing. In 2021, UNHCR and its partners organized trainings in such interventions for 361 staff in refugee camps in Angola, Bangladesh, Cameroon, DRC, Ethiopia, Jordan, Kenya, Mauritania, Niger, Nigeria, Republic of Congo, Rwanda, Tanzania, and Uganda (UNHCR Public Health Section, oral communication, March 2022).
3. Integration of mental health and psychosocial support into community health work. This includes training community health workers and other community volunteers in the identification and follow-up of people with severe or complex mental health conditions, and training community health workers in basic psychosocial skills, including the provision of Psychological First Aid.

Beyond these actions, it is clear that additional research and investment are needed to address neglected issues such as substance use and suicide prevention [53, 65]. Notably, a toolkit to address substance use in humanitarian settings is expected to be released in 2022 by the United Nations Office on Drugs and Crime with support from UNHCR and WHO. Furthermore, UNHCR will release the following new guidance in 2022: *Planning for Suicide Prevention and Mitigation in Refugee Settings: A Toolkit for Multisectoral Action*.

These activities by UNHCR fit within major efforts by a range of organizations to strengthen mental health and psychosocial support in humanitarian settings. Importantly, a major new development is the *Mental Health and Psychosocial Support Minimum Services Package* by UNHCR, WHO, and other collaborating agencies (www.mhpssmsp.org). This multi-sectoral package describes key actions needed to improve mental health and well-being among conflict-affected populations by fully integrating mental health and psychosocial support services into health, education, and protection activities.

This analysis of 10 years of MNS consultations in refugee primary health care settings underscores that more needs to be done to enable primary health care services to address the needs of refugees with MNS disorders. Overall, this requires sustained investments into supportive clinical training and supervision of primary health care workers, and increased efforts to ensure that refugees have access to a wider range of mental health and psychosocial support services within community settings.

## Supplementary Information

**Additional file 1.**

## Data Availability

The data that support the findings of this study are available from UNHCR, but restrictions apply to the availability of these data, which were used under license for the current study, and so are not publicly available.

## Abbreviations

CAR: Central African Republic
DRC: Democratic Republic of Congo
HIG: Humanitarian Intervention Guide
HIS: Health information system
IASC: Inter-Agency Standing Committee
iRHIS: Integrated Refugee Health Information System
LMIC: Low- and middle-income country
mhGAP: Mental Health Gap Action Programme
MNS: Mental, neurological, and substance use
PTSD: Post-traumatic stress disorder
UNHCR: United Nations High Commissioner for Refugees
WHO: World Health Organization

## References

[1] UNHCR (2021). Global trends: forced displacement in 2020.

[2] Charlson F, van Ommeren M, Flaxman A, Cornett J, Whiteford H, Saxena S (2019). New WHO prevalence estimates of mental disorders in conflict settings: a systematic review and meta-analysis. Lancet..

[3] Lindert J, von Ehrenstein OS, Priebe S, Mielck A, Brähler E (1982). Depression and anxiety in labor migrants and refugees--a systematic review and meta-analysis. Soc Sci Med.

[4] Reed RV, Fazel M, Jones L, Panter-Brick C, Stein A (2012). Mental health of displaced and refugee children resettled in low-income and middle-income countries: risk and protective factors. Lancet..

[5] Steel Z, Chey T, Silove D, Marnane C, Bryant RA, van Ommeren M (2009). Association of torture and other potentially traumatic events with mental health outcomes among populations exposed to mass conflict and displacement: a systematic review and meta-analysis. JAMA..

[6] Vos T, Abajobir AA, Abate KH, Abbafati C, Abbas KM, Abd-Allah F (2017). Global, regional, and national incidence, prevalence, and years lived with disability for 328 diseases and injuries for 195 countries, 1990–2016: a systematic analysis for the Global Burden of Disease Study 2016. Lancet..

[7] Silove D, Ventevogel P, Rees S (2017). The contemporary refugee crisis: an overview of mental health challenges. World Psychiatry.

[8] Ngugi AK, Bottomley C, Kleinschmidt I, Sander JW, Newton CR (2010). Estimation of the burden of active and life-time epilepsy: a meta-analytic approach. Epilepsia..

[9] Mateen FJ, Carone M, Nyce S, Ghosn J, Mutuerandu T, Al-Saedy H (2012). Neurological disorders in Iraqi refugees in Jordan: data from the United Nations Refugee Assistance Information System. J Neurol..

[10] Meyer A-C, Dua T, Ma J, Saxena S, Birbeck G (2010). Global disparities in the epilepsy treatment gap: a systematic review. Bull World Health Organ..

[11] Jones L, Asare JB, El Masri M, Mohanraj A, Sherief H, van Ommeren M (2009). Severe mental disorders in complex emergencies. Lancet Lond Engl..

[12] Silove D, Bateman CR, Brooks RT, Fonseca CAZ, Steel Z, Rodger J (2008). Estimating clinically relevant mental disorders in a rural and an urban setting in postconflict Timor Leste. Arch Gen Psychiatry..

[13] Brandt L, Henssler J, Müller M, Wall S, Gabel D, Heinz A. Risk of psychosis among refugees: a systematic review and meta-analysis. JAMA Psychiatry. 2019. 10.1001/jamapsychiatry.2019.1937. 10.1001/jamapsychiatry.2019.1937 PMC6694397 31411649

[14] Nygaard M, Sonne C, Carlsson J (2017). Secondary psychotic features in refugees diagnosed with post-traumatic stress disorder: a retrospective cohort study. BMC Psychiatry..

[15] Rathke H, Poulsen S, Carlsson J, Palic S (2020). PTSD with secondary psychotic features among trauma-affected refugees: The role of torture and depression. Psychiatry Res..

[16] Ezard N (2012). Substance use among populations displaced by conflict: a literature review. Disasters..

[17] Horyniak D, Melo JS, Farrell RM, Ojeda VD, Strathdee SA (2016). Epidemiology of substance use among forced migrants: a global systematic review. PloS One..

[18] Jones L, Ventevogel P (2021). From exception to the norm: how mental health interventions have become part and parcel of the humanitarian response. World Psychiatry.

[19] Silove D (2021). Challenges to mental health services for refugees: a global perspective. World Psychiatry..

[20] Inter-Agency Standing Committee (2007). IASC Guidelines on Mental Health and Psychosocial Support in Emergency Settings.

[21] World Health Organization, United Nations High Commissioner for Refugees (2015). mhGAP Humanitarian Intervention Guide (mhGAP-HIG): clinical management of mental, neurological and substance use conditions in humanitarian emergencies.

[22] Echeverri C, Le Roy J, Worku B, Ventevogel P. Mental health capacity building in refugee primary health care settings in Sub-Saharan Africa: impact, challenges and gaps. Glob Ment Health C. 2018;5:e28. 10.1017/gmh.2018.19 PMC6128042 30202535

[23] Hughes P, Hijazi Z, Saeed K (2016). Improving access to mental healthcare for displaced Syrians: case studies from Syria, Iraq and Turkey. BJPsych Int.

[24] Momotaz H, Ahmed HU, Uddin MMJ, Karim R, Khan MA, Al-Amin R (2019). Implementing the mental health gap action programme in Cox’s Bazar, Bangladesh. Intervention.

[25] Sherchan S, Samuel R, Marahatta K, Anwar N, Van Ommeren MH, Ofrin R (2017). Post-disaster mental health and psychosocial support: experience from the 2015 Nepal earthquake. WHO South East Asia J Public Health..

[26] Tarannum S, Elshazly M, Harlass S, Ventevogel P (2019). Integrating mental health into primary health care in Rohingya refugee settings in Bangladesh: experiences of UNHCR. Intervention..

[27] Ryan G, De Silva M, Terver JS, Ochi OP, Eaton J (2015). Information systems for global mental health. Lancet Psychiatry..

[28] Haskew C, Spiegel P, Tomczyk B, Cornier N, Hering H (2010). A standardized health information system for refugee settings: rationale, challenges and the way forward. Bull World Health Organ..

[29] Ventevogel P, Ryan GK, Kahi V, Kane JC (2019). Capturing the essential: revising the mental health categories in UNHCR’s Refugee Health Information System. Intervention..

[30] Kane JC, Ventevogel P, Spiegel P, Bass JK, van Ommeren M, Tol WA (2014). Mental, neurological, and substance use problems among refugees in primary health care: analysis of the Health Information System in 90 refugee camps. BMC Med..

[31] UNHCR. Refugee Data Finder. Refugee Data Finder. 2020. https://www.unhcr.org/refugee-statistics/. Accessed 31 Jul 2020.

[32] UNHCR (2019). Global trends: forced displacement in 2018.

[33] De Silva MJ, Lee L, Fuhr DC, Rathod S, Chisholm D, Schellenberg J (2014). Estimating the coverage of mental health programmes: a systematic review. Int J Epidemiol..

[34] StataCorp. (2015). Stata Statistical Software: Release 14.

[35] Hughes P, Thomson S (2019). mhGAP – the global scenario. Prog Neurol Psychiatry..

[36] van Ommeren M, Hanna F, Weissbecker I, Ventevogel P (2015). Mental health and psychosocial support in humanitarian emergencies. East Mediterr Health J.

[37] Karaoğlan Kahiloğulları A, Alataş E, Ertuğrul F, Malaj A (2020). Responding to mental health needs of Syrian refugees in Turkey: mhGAP training impact assessment. Int J Ment Health Syst..

[38] Faregh N, Lencucha R, Ventevogel P, Dubale BW, Kirmayer LJ (2019). Considering culture, context and community in mhGAP implementation and training: challenges and recommendations from the field. Int J Ment Health Syst..

[39] Humayun A, Haq I, Khan FR, Azad N, Khan MM, Weissbecker I. Implementing mhGAP training to strengthen existing services for an internally displaced population in Pakistan. Glob Ment Health. 2017;4:e6. 10.1017/gmh.2017.1 PMC5454790 28596907

[40] Pérez-Sales P, Férnandez-Liria A, Baingana F, Ventevogel P (2011). Integrating mental health into existing systems of care during and after complex humanitarian emergencies: rethinking the experience. Intervention..

[41] Nguyen AJ, Rykiel N, Murray L, Amin A, Haroz E, Lee C (2019). Stakeholder perspectives on integration of mental health services into primary care: a mixed methods study in Northern Iraq. Int J Ment Health Syst..

[42] Schubert J (2018). Mental health & psychosocial support for Syrian refugees in Jordan – a capacity analysis of the national implementation of WHO’s mhGAP.

[43] Ventevogel P, Jordans M, Reis R, de Jong J (2013). Madness or sadness? Local concepts of mental illness in four conflict-affected African communities. Confl Health..

[44] Andersson LMC, Schierenbeck I, Strumpher J, Krantz G, Topper K, Backman G (2013). Help-seeking behaviour, barriers to care and experiences of care among persons with depression in Eastern Cape, South Africa. J Affect Disord.

[45] Menberu M, Mekonen T, Azale T, Ayano G, Yimer S, Getnet A (2018). Health care seeking behavior for depression in Northeast Ethiopia: depression is not considered as illness by more than half of the participants. Ann Gen Psychiatry..

[46] Nsereko JR, Kizza D, Kigozi F, Ssebunnya J, Ndyanabangi S, Flisher AJ (2011). Stakeholder’s perceptions of help-seeking behaviour among people with mental health problems in Uganda. Int J Ment Health Syst..

[47] Tay AK, Riley A, Islam R, Welton-Mitchell C, Duchesne B, Waters V (2019). The culture, mental health and psychosocial wellbeing of Rohingya refugees: a systematic review. Epidemiol Psychiatr Sci..

[48] Bolton P, Lee C, Haroz EE, Murray LK, Dorsey S, Robinson C (2014). A transdiagnostic community-based mental health treatment for comorbid disorders: development and outcomes of a randomized controlled trial among Burmese refugees in Thailand. PLoS Med..

[49] Greene MC, Likindikoki S, Rees S, Bonz A, Kaysen D, Misinzo L (2021). Evaluation of an integrated intervention to reduce psychological distress and intimate partner violence in refugees: Results from the Nguvu cluster randomized feasibility trial. PloS One..

[50] Tol WA, Leku MR, Lakin DP, Carswell K, Augustinavicius J, Adaku A (2020). Guided self-help to reduce psychological distress in South Sudanese female refugees in Uganda: a cluster randomised trial. Lancet Glob Health..

[51] Tay AK, Miah MAA, Khan S, Mohsin M, Alam AM, Ozen S, et al. A naturalistic evaluation of group integrative adapt therapy (IAT-G) with Rohingya refugees during the emergency phase of a mass humanitarian crisis in Cox's Bazar, Bangladesh. EClinicalMedicine. 2021;38:100999. 10.1016/j.eclinm.2021.100999 PMC8413262 34505027

[52] Acarturk C, Uygun E, Ilkkursun Z, Yurtbakan T, Kurt G, Adam-Troian J (2022). Group problem management plus (PM+) to decrease psychological distress among Syrian refugees in Turkey: a pilot randomised controlled trial. BMC Psychiatry..

[53] Greene MC, Ventevogel P, Kane JC (2019). Substance use services for refugees. Bull World Health Organ..

[54] Kane JC, Greene MC (2018). Addressing alcohol and substance use disorders among refugees: a desk review of intervention approaches.

[55] Roberts B, Ezard N (2015). Why are we not doing more for alcohol use disorder among conflict-affected populations?. Addict Abingdon Engl..

[56] Chung RC, Bemak F, Kagawa-Singer M (1998). Gender differences in psychological distress among Southeast Asian refugees. J Nerv Ment Dis..

[57] Kamau M, Silove D, Steel Z, Catanzaro R, Bateman C, Ekblad S (2004). Psychiatric disorders in an African refugee camp. Intervention.

[58] Weissbecker I, Hanna F, El Shazly M, Gao J, Ventevogel P, Wenzel T, Drožđek B (2019). Integrative mental health and psychosocial support interventions for refugees in humanitarian crisis settings. An Uncertain Safety: Integrative Health Care for the 21st Century Refugees.

[59] Barbui C, Purgato M, Abdulmalik J, Acarturk C, Eaton J, Gastaldon C (2020). Efficacy of psychosocial interventions for mental health outcomes in low-income and middle-income countries: an umbrella review. Lancet Psychiatry..

[60] Haroz EE, Nguyen AJ, Lee CI, Tol WA, Fine SL, Bolton P (2020). What works in psychosocial programming in humanitarian contexts in low- and middle-income countries: a systematic review of the evidence. Intervention..

[61] UNHCR (2013). Operational guidance: mental health & psychosocial support programming for refugee operations.

[62] UNHCR (2021). Strengthening mental health and psychosocial support in 2021.

[63] UNHCR (2021). UNHCR Global Strategy for Public Health 2021-2025.

[64] Bryant RA, Bawaneh A, Awwad M, Al-Hayek H, Giardinelli L, Whitney C (2022). Effectiveness of a brief group behavioral intervention for common mental disorders in Syrian refugees in Jordan: A randomized clinical trial. PLoS Med..

[65] Haroz EE, Decker E, Lee C, Bolton P, Spiegel P, Ventevogel P (2020). Evidence for suicide prevention strategies with populations in displacement: a systematic review. Intervention (Amstelveen).

