# Supplementary material for: Ten years of tracking mental health in refugee primary health care settings: an updated analysis of data from UNHCR’s Health Information System (2009–2018)
Source: BMC Med. 2022 May 16;20:183. doi: 10.1186/s12916-022-02371-8 (PMC9109385; doi:10.1186/s12916-022-02371-8)
Supplement: Supplementary file 1 — Additional file 1. [file 12916_2022_2371_MOESM1_ESM.pdf]

# Health Information System

Reporting Form

Organisation: ______________

Location: ______________

Reporting period: ______________

## 3.0 Morbidity

### 3.6 Mental illness

(enter New and Revisits)

| | Refugee | | | | | | | | National | |
|---|---|---|---|---|---|---|---|---|---|---|
| | 0 - 4 | | 5 - 17 | | 18 - 59 | | ≥ 60 | | | |
| | M | F | M | F | M | F | M | F | M | F |
| 1. Epilepsy / seizures | | | | | | | | | | |
| 2. Alcohol or other substance use disorder | | | | | | | | | | |
| 3. Mental retardation / intellectual disability | | | | | | | | | | |
| 4. Psychotic disorder | | | | | | | | | | |
| 5. Severe emotional disorder | | | | | | | | | | |
| 6. Other psychological complaint | | | | | | | | | | |
| 7. Medically unexplained somatic complaint | | | | | | | | | | |
